# Multicenter evaluation of the Selux Next-Generation Phenotyping antimicrobial susceptibility testing system

**DOI:** 10.1128/jcm.00546-23

**Published:** 2023-12-05

**Authors:** Kristin R. Baker, Kelly Flentie, Benjamin R. Spears, Sergey Mozharov, Kristen Roberts, Asmae El ganbour, Mark Somers, John Calkwood, Jamie Liu, Kayla DaPonte, Nikitha Sam, Gurleen Kaur, Felicia Chen, Jonathan Donato, Alan Chao, Autumn Lewis, Jingzi Sherman, Karen Mortimer, Amanda T. Harrington, Maria Traczewski, Darcie Carpenter, Dee Shortridge, Jill Lindley, Alexander Diep, Emmet Norton, Matt Green, Joe Gajewski, Rebecca Landrith, Fatuma Nalubega, Justin McCallum, Melissa Beiswenger, Brittany Dolan, Kathleen Brennan, Afton Carpenter, Aleksandar Vacic, Alec N. Flyer, Virginia M. Pierce, David C. Hooper, James S. Lewis II, Eric Stern

**Affiliations:** 1 Selux Diagnostics, Charlestown, Massachusetts, USA; 2 Pathology and Laboratory Medicine, Loyola University Medical Center, Maywood, Illinois, USA; 3 Clinical Microbiology Institute (CMI), Wilsonville, Oregon, USA; 4 IHMA, Schaumburg, Illinois, USA; 5 JMI Laboratories/Element, North Liberty, Iowa, USA; 6 Element, Acton, Massachusetts, USA; 7 Boston Analytical, Salem, New Hampshire, USA; 8 Pathology, University of Michigan Medical School, Ann Arbor, Michigan, USA; 9 Medicine, Massachusetts General Hospital, Boston, Massachusetts, USA; 10 Department of Pharmacy Services, Oregon Health and Science University Hospitals and Clinics, Portland, Oregon, USA; Johns Hopkins University, Baltimore, Maryland, USA

**Keywords:** antimicrobial susceptibility testing, clinical diagnostics, clinical microbiology

## Abstract

The Selux Next-Generation Phenotyping (NGP) system (Charlestown, MA) is a new antimicrobial susceptibility testing system that utilizes two sequential assays performed on all wells of doubling dilution series to determine MICs. A multicenter evaluation of the performance of the Selux NGP system compared with reference broth microdilution was conducted following FDA recommendations and using FDA-defined breakpoints. A total of 2,488 clinical and challenge isolates were included; gram-negative isolates were tested against 24 antimicrobials, and gram-positive isolates were tested against 15 antimicrobials. Data is provided for all organism-antimicrobial combinations evaluated, including those that did and did not meet FDA performance requirements. Overall very major error and major error rates were less than 1% (31/3,805 and 107/15,606, respectively), essential agreement and categorical agreement were >95%, reproducibility was ≥95%, and the average time-to-result (from time of assay start to time of MIC result) was 5.65 hours.

## INTRODUCTION

Overuse of broad-spectrum antibiotics may result in worse patient outcomes and contribute to the current antibiotic resistance crisis (1–4). The use of broad-spectrum empiric antibiotics is driven in part by the delay in reporting antimicrobial susceptibility test (AST) results in today’s standard of care workflows (5–9). While bacterial identification often occurs within a day of specimen collection due to recent technological advances in multiplex PCR and mass spectrometry, AST results often take substantially longer (5–13).

Commercial AST systems are common in US clinical laboratories and are crucial to patient care. However, current AST systems require improvement in three key areas: speed; the number of drugs that can be run on a single panel (the “menu”), including the rapid addition of new antimicrobials; and the implementation of up-to-date breakpoints (6–9, 14, 15). An AST platform that provides rapid results from broad antibiotic menus with updated breakpoints would help address these issues. While multiple rapid AST platforms are in various stages of development, many lack the ability to perform AST on a variety of clinical isolates (6).

We previously described a bulk fluorescent assay that produces MIC determinations by measuring bacterial surface area (16). An extension of that method allowed for the development of a high-throughput, random-access, automated AST system [Selux Next-Generation Phenotyping (NGP) System, Charlestown, MA]. Here, we describe the accuracy, reproducibility, and time-to-results (TTRs) of this system in a multicenter evaluation.

## MATERIALS AND METHODS

### Bacterial strains, isolates, and routine culture methods

Bacterial isolates included 706 clinical and 159 challenge gram-positive (GP) isolates, 1,401 clinical and 222 challenge gram-negative (GN) isolates, and 6 quality control strains. To ensure geographic diversity, clinical isolates from across the U.S. were provided by four sites: Loyola University (Chicago, IL), Clinical Microbiology Institute (Wilsonville, OR), International Health Management Associates (IHMA, Schaumburg, IL), and JMI Laboratories (North Liberty, IA). 27% of isolates were tested on the Selux NGP system within 6 months of being isolated from the patient. The remainder of the clinical isolates was stocks from routine clinical specimens stored at each site. No more than one isolate per species per patient was tested. Challenge isolates were composed of organisms sourced from multiple laboratories. Each clinical and challenge isolate was prepared in triplicate as frozen glycerol stocks; one set was shipped to the testing site, the second to JMI Laboratories (reference laboratory), and a third was stored at the collection/source site. Organism identifications determined by the four collection sites were confirmed by the reference laboratory, and any samples with mismatching identifications were excluded from the analysis. Organism identifications were provided to sites performing AST. Organisms were routinely subcultured twice overnight at 35°C prior to testing.

### Multicenter evaluation study

The Selux NGP system [Selux Diagnostics (Charlestown, MA)] was evaluated in a multicenter study from 2020 to 2022. Following FDA guidance (17), clinical, challenge, reproducibility, and quality control isolates were processed on the investigational use only (IUO) Selux NGP system installed at three sites: Selux Diagnostics (Charlestown, MA), Analytical Laboratory Group (purchased by Element Materials Technology, Acton, MA), and Boston Analytical (Salem, NH). All sites used separate inocula and were blinded to all clinical and challenge isolate reference results, which were determined by broth microdilution (BMD) testing at an independent reference laboratory. Repeat testing of isolates on the Selux NGP system was only performed if there was a failure in the system (quality control failure or mechanical failure) or the sample failed purity testing (as described below).

The multicenter study evaluated the system using 15 GP antimicrobials (including one screening test) and 24 GN antimicrobials. FDA Susceptibility Test Interpretive Criteria breakpoints from June 2021 were applied in the analysis of system performance. All study results were submitted to FDA for 510(k) clearance following the FDA’s Special Controls guidance for AST systems (17) and the Selux NGP system received FDA-clearance on 18 January 2023 and 19 April 2023 for the GP and GN panels, respectively. The multicenter study results described here are the same as those provided to FDA, except where limitations imposed by FDA upon granting 510(k) clearance have been applied to the data. The TTRs, defined as the time panels placed on the analyzer (Selux NGP assay start) until an MIC value was generated, were also evaluated during the multicenter study by calculating the mean, minimum, and maximum TTR for each species.

Selux NGP system quality control was performed daily during testing at all laboratories. Subculture plates were prepared from all sample inocula, including quality control, and read for organism purity. Quantitative cultures from sample inocula were performed on suspensions for all quality control organisms and for 10% of clinical and challenge isolates.

An inter-site reproducibility study was performed using GP and GN panels at all three testing locations, and one site (Selux Diagnostics, Charlestown, MA) performed intra-site testing. The inter-site reproducibility study evaluated a minimum of 25 isolates with on-scale MICs including 104 staphylococci, 44 enterococci, 202 Enterobacterales, 4 *Acinetobacter baumannii*, and 20 *Pseudomonas aeruginosa* species. Each isolate was tested once at each of the three sites using 34 antimicrobials resulting in a minimum of 75 results per antimicrobial. The intra-site reproducibility study evaluated a minimum of five isolates in triplicate for each antimicrobial, including 12 staphylococci, 5 enterococci, 19 Enterobacterales, 1 *A. baumannii*, and 3 *P. aeruginosa* species. Results were evaluated as best- and worst-case reproducibility for all antimicrobials. Best-case performance assumes off-scale results were within essential agreement (EA), and worst-case performance assumes off-scale results were not within EA.

#### BMD method

BMD was performed following CLSI M07 guidelines (18). BMD was performed by JMI Laboratories in triplicate using three separate inocula (17). For cases where triplicate results had a mode or where the median results were in EA with each of the two other results, the mode or median MIC, respectively, was used as the comparator. In cases where these criteria were not met (<10% occurrence), two additional replicates were tested from separate inocula, and the median MIC of the five datapoints was used.

#### Selux NGP system panels

Panels were prepared using American National Standards Institute (ANSI, Washington DC) 384-well standard microtiter plates containing dried antimicrobials sealed with desiccant and stored at ambient temperature until use. GP panels contain 15 antibiotics, and GN panels contain 24 antibiotics. Only the organism Gram type (GP or GN) is required prior to AST processing; however, species identification is necessary for AST results reporting. FDA-cleared antimicrobial agents and combinations present on the panels are listed in Table S1.

#### System sample processing

All isolates were processed according to the manufacturer’s IUO instructions. The Selux NGP system comprises three stations: a sample preparation station and computer, the automated inoculator, and the analyzer (Fig. S1). A reusable carrier is used to manually transfer panels between each station. At the sample preparation station, users prepared 0.4–0.6 McFarland bacterial inocula in saline (2.5–3.5 mL volume) and placed them on the carrier with a corresponding AST panel. Up to four samples may be loaded on a carrier before loading into the inoculator. The inoculator diluted the inoculum into cation-adjusted Mueller-Hinton broth (1:200 dilution) and added 50 µL to panel wells. After inoculation was complete, carriers were manually loaded into the analyzer, a random-access system with a 96-panel capacity that automates all remaining AST assay steps. When organism identification is available, the system applies the appropriate algorithm for each drug-bacteria combination to determine the MIC as well as the interpretive criteria results.

### Selux NGP system assays

All assays are automated within the Selux NGP system. Each is detailed below.

#### Growth assay

This assay gates the initiation of the AST assays (see following) by ensuring sufficient bacterial growth has been achieved in antibiotic-containing wells before AST assay probes are added. The growth assay uses viability signals and optical density information in antibiotic-free positive control wells to determine if a sufficient growth threshold has been achieved. The threshold is a normalized value of the positive control (nutrient media and organism without antibiotic) to the negative control (nutrient media only) using viability (see following) or optical density signal measurements. For both GN and GP organisms, panels were incubated 3 hours prior to the first measurement of growth in the positive control wells using a plate reader. If sufficient growth was not achieved, the panel was returned to the incubator for an additional 90 minutes. This step was repeated until sufficient growth was achieved for each panel.

#### Viability assay

After sufficient growth was achieved, a viability dye formulation was added to all wells on the panel. Panels were incubated for an hour to allow microorganisms to reduce the non-fluorescent resazurin dye to a fluorescent product, resorufin. Methylene blue serves as an intermediate electron transfer agent, bridging the energy level gap between resazurin and the reductive cellular respiration machinery (19, 20). The GN formulation additionally contains 1-methoxy-5-methylphenazinium methyl sulfate (1-methoxy PMS) as an additional electron transfer agent to enhance resazurin reduction. After the incubation period, the resorufin fluorescence in each well was determined.

#### Surface area assay

Following viability assay completion, GP and GN bacteria that exhibited growth at a colistin concentration of 8 µg/mL in the viability assay were treated with cetrimonium bromide to increase probe binding efficiency. All other bacteria were treated with phosphate-buffered saline containing Tween 20. Panels were then centrifuged to pellet bacteria, and all growth media, antibiotics, and reagents were removed. A europium chelate probe (16) was then added to each well, and the panels were shaken at room temperature to promote electrostatic binding. The probe “head” is a divalent cation that adheres to anionic microorganism surfaces, and the probe “tail” is a europium chelate that exhibits fluorescence intensity through time-resolved fluorescence (21). After probe binding, one wash cycle removed the unbound probe. Microorganism pellets were then resuspended in phosphate-buffered saline containing Tween 20, and time-resolved fluorescence was measured in each well.

## RESULTS

Multicenter study results for GP and GN organisms are presented in Tables 1 and 2. EAs were above 90% for all organism-antibiotic combinations tested, except, *P. aeruginosa* with ceftazidime-avibactam (89.6%). Similarly, categorical agreements were above 90% for all organism-antibiotic combinations tested except Enterobacterales with ampicillin-sulbactam, cefazolin, and cefoxitin and *P. aeruginosa* for levofloxacin. In each of these instances, the EA exceeded the FDA minimum EA requirements of 90%, at 97.6%, 95.1%, 94%, and 93.9%, respectively (17). Overall very major error (VME) rates were 0.82% (24/2,934) and 0.80% (7/871) for GN and GP, respectively. Overall major error (ME) rates for GN and GP were 0.73% (91/12,539) and 0.52% (16/3067), respectively. Individual VME rates were below 2% for all combinations, except for a single error in the only daptomycin-resistant *Staphylococcus aureus*, *S. aureus* with penicillin (2.5%), Enterobacterales with minocycline (4.9%) and meropenem-vaborbactam (2.4%), and Enterobacterales and *P. aeruginosa* with piperacillin-tazobactam (2.5% and 7.2%, respectively). Individual ME rates were at or below 3%, except *A. baumannii* with ampicillin-sulbactam (3.1%), meropenem (3.2%), and minocycline (3.3%).

**TABLE 1:** Results for GP organisms using the Selux NGP system^*a*^^,*b*^

Antimicrobial	Organism group^*c*^	Total tested	EA		CA		R		VME		ME		mE	
			#	%^*d*^	#	%^*d*^	#	%^*d*^	#	%^*d*^	#	%^*d*^	#	%^*d*^
Ampicillin	*Enterococcus* spp. EFS and EFM	299	294	98.3	299	100	163	54.5	0	0.0	0	0.0	0	0.0
Clindamycin	*Staphylococcus* spp. SA and SE	195	187	95.9	192	98.5	48	24.6	0	0	1	0.7	2	1.0
Ceftaroline	*Staphylococcus* spp. SA	138	136	98.6	136	98.6	1	0.7	0	0.0	1	0.7	1	0.7
Daptomycin	*Enterococcus* spp. EFS	116	109	94	116	100	0	0.0	0	N/A	0	0.0	0	0.0
	*Staphylococcus* spp. SA	134	132	98.5	133	99.3	1	0.7	1	100	0	0.0	0	0.0
Delafloxacin	*Enterococcus* spp. EFS	180	175	97.2	164	91.1	38	21.1	0	0.0	2	1.6	14	7.8
	*Staphylococcus* spp. SA and SH	222	220	99.1	215	96.8	12	5.4	0	0.0	0	0.0	7	3.2
Eravacycline	*Enterococcus* spp. EFS	114	107	93.9	114	100	1	0.9	0	0.0	0	0.0	0	0.0
	*Staphylococcus* spp. SA	118	118	100	118	100	2	1.7	0	0.0	0	0.0	0	0.0
Erythromycin	*Staphylococcus* spp. SA	145	141	97.2	141	97.2	80	55.2	0	0.0	0	0.0	4	2.8
Levofloxacin	*Enterococcus* spp. EFS and EFM	275	268	97.5	270	98.2	127	46.2	0	0.0	2	1.4	3	1.1
	*Staphylococcus* spp. SA	135	132	97.8	130	96.3	43	31.9	0	0.0	1	1.2	4	3.0
Linezolid	*Enterococcus* spp. EFS and EFM	299	287	96	294	98.3	1	0.3	0	0.0	3	1.0	2	0.7
	*Staphylococcus* spp. SA, SE, and SH	165	163	98.8	164	99.4	3	1.8	0	0.0	1	0.6	0	0.0
Minocycline	*Staphylococcus* spp. SA	113	113	100	113	100	0	0.0	0	N/A	0	0.0	0	0.0
Oxacillin	*S. aureus*, *Staphylococcus lugdunensis*	160	149	93.1	158	98.8	52	32.5	1	1.9	1	0.9	0	0.0
Penicillin	*Enterococcus* spp. EFS and EFM	131	119	90.8	130	99.2	1	0.8	0	0.0	1	0.8	0	0.0
	*Staphylococcus* spp. SA	204	186	91.2	200	98	163	79.9	4	2.5	0	0.0	0	0.0
Trimethoprim	*Staphylococcus* spp. SA, SC, SH, SS, and SSM	156	153	98.1	156	100	14	9.0	0	0.0	0	0.0	0	0.0
Vancomycin	*Enterococcus* spp. EFS and EFM	173	164	94.8	171	98.8	40	23.1	0	0.0	0	0.0	2	1.2
	*S. aureus*	238	236	99.2	236	99.2	0	0	0	N/A	1	0.4	1	0.4
	Coagulase-negative *Staphylococcus* spp. SC, SCH, SE, SH, SHM, SI, SL, SS, SSF, and SSM	111	109	98.2	111	100	0	0	0	N/A	0	0.0	0	0.0

Screening test	Organism group	Total tested	Agree		R		VME				ME			
			#	%^*d*^	#	%^*d*^	#	%^*d*^			#	%^*d*^		
Cefoxitin screen	*S. aureus*, *S. lugdunensis*	175	172	98.3	81	46.3	1	1.2			2	2.1		

^*a*^ CA, categorical agreement; R, resistant isolates; mE, minor errors.

^*b*^ Refer to Supplementary table 1 for Selux NGP system FDA-cleared species and antimicrobial agents.

^*c*^ Organism group includes species: EFS*, Enterococcus faecalis*; EFM, *Enterococcus faecium*; SA, *Staphylococcus aureus*; SC, *Staphylococcus capitis*; SCH, *Staphylococcus cohnii*; SE, *Staphylococcus epidermidis*; SH, *Staphylococcus haemolyticus*; SHM, *Staphylococcus hominis*; SI, *Staphylococcus intermedius group*; SL, *Staphylococcus lugdunensis*; SS, *Staphylococcus saprophyticus*; SSF, *Staphylococcus schleiferi*; SSM, *Staphylococcus simulans*.

^*d*^ Percentage calculated as follows: %VME = total number VME/total number resistant isolates × 100; %ME = total number ME/total number susceptible isolates × 100; %mE = total number mE/total number isolates × 100.

**TABLE 2:** Results for GN organisms using the Selux NGP system^*a*^^,*b*^

Antimicrobial	Organism Group^*c*^	Total tested	EA		CA		R		VME		ME		mE	
			#	%^*d*^	#	%^*d*^	#	%^*d*^	#	%^*d*^	#	%^*d*^	#	%^*d*^
Amikacin	*P. aeruginosa*	165	150	90.9	162	98.2	7	4.2	0	0.0	0	0.0	3	1.8
Amoxicillin-clavulanate	Enterobacterales CK, EC, KP, KV, KO, PM, and PV	593	564	95.1	550	92.7	111	18.7	1	0.9	5	1.2	37	6.2
Ampicillin	Enterobacterales EC and PM	250	238	95.2	248	99.2	117	46.8	0	0.0	1	0.8	1	0.4
Ampicillin-sulbactam	*A. baumannii* complex	123	113	91.9	114	92.7	52	42.3	1	1.9	2	3.1	6	4.9
	Enterobacterales EC, KP, KV, KO, PM, and PV	493	481	97.6	424	86	148	30.0	0	0.0	0	0.0	69	14.0
Aztreonam	Enterobacterales EC	183	179	97.8	178	97.3	46	25.1	0	0.0	0	0.0	5	2.7
Cefazolin	Enterobacterales CK, EC, KP, KV, and PM	412	392	95.1	308	74.8	218	52.9	1	0.5	1	1.0	102	24.8
Cefepime	Enterobacterales CF, CK, ECC, EC, KP, KV, KA, KO, MM, PM, PV, and SM	953	905	95	917	96.2	126	13.2	2	1.6	8	1.0	26	2.7
	*P. aeruginosa*	173	162	93.6	160	92.5	30	17.3	0	0.0	4	2.8	0	0.0
Cefoxitin	Enterobacterales CK, EC, KP, KV, KO, MM, PM, and PV	605	569	94	492	81.3	105	17.4	2	1.9	9	2.4	102	16.9
Ceftazidime	*A. baumannii complex*	127	116	91.3	120	94.5	57	44.9	0	0.0	0	0.0	7	5.5
	Enterobacterales CF, CK, ECC, EC,KP, KV,KA, KO, PM, PV, and SM	787	752	95.6	759	96.4	208	26.4	1	0.5	1	0.2	26	3.3
	*P. aeruginosa*	96	89	92.7	94	97.9	18	18.8	0	0.0	2	2.6	0	0.0
Ceftazidime-avibactam	Enterobacterales CF, CK, ECC, EC, KP, KV, KA, KO, MM, PM, PV, PA, and SM	815	793	97.3	810	99.4	32	3.9	0	0.0	5	0.6	0	0.0
	*P. aeruginosa*	163	146	89.6	158	96.9	10	6.1	0	0.0	2	1.3	0	0.0
Ceftriaxone	Enterobacterales CF, CK, ECC, EC, KP, KV, KA, KO, PM, and SM	679	664	97.8	671	98.8	185	27.2	1	0.5	4	0.8	3	0.4
Ciprofloxacin	Enterobacterales CF, CK,ECC ,EC ,KP ,KV ,KA ,KO, MM, PM, PV, and SM	788	766	97.2	759	96.3	175	22.2	0	0.0	3	0.5	26	3.3
	*P. aeruginosa*	169	162	95.9	160	94.7	33	19.5	0	0.0	4	3.0	5	3.0
Eravacycline	Enterobacterales CF, ECC, EC, KP, KV, and KO	383	377	98.4	378	98.7	52	13.6	0	0.0	1	0.3	0	0.0
Ertapenem	Enterobacterales CF, CK, ECC, EC, KP, KV, KA, KO, MM, PM, PV, and SM	952	927	97.4	937	98.4	106	11.1	0	0.0	5	0.6	10	1.1
Gentamicin	Enterobacterales CF, CK, ECC, EC, KP, KV, KA, KO, MM, PM, PV, and SM	817	788	96.5	807	98.8	121	14.8	2	1.7	1	0.1	7	0.9
	*P. aeruginosa*	209	188	90	203	97.1	15	7.2	0	0.0	0	0.0	6	2.9
Imipenem	*A. baumannii complex*	123	116	94.3	123	100	60	48.8	0	0.0	0	0.0	0	0.0
Imipenem-Relebactam	Enterobacterales CF, CK, EC, KP, KV, KA, and KO	463	447	96.5	459	99.1	32	6.9	0	0.0	2	0.5	2	0.4
	*P. aeruginosa*	165	159	96.4	162	98.2	6	3.6	0	0.0	0	0.0	3	1.8
Levofloxacin	Enterobacterales CF, CK, ECC, EC, KP, KV, KA, KO, MM, PM, PV, and SM	780	752	96.4	744	95.4	161	20.6	1	0.6	3	0.5	32	4.1
	*P. aeruginosa*	98	92	93.9	86	87.8	40	40.8	0	0.0	1	2.0	11	11.2
Meropenem	*A. baumannii* complex	126	119	94.4	124	98.4	63	50.0	0	0.0	2	3.2	0	0.0
	Enterobacterales CF, CK, ECC, EC, KP, KV, KA, KO, MM, PM, PV, and SM	853	828	97.1	843	98.8	79	9.3	1	1.3	5	0.6	4	0.5
	*P. aeruginosa*	175	163	93.1	168	96	38	21.7	0	0.0	0	0.0	7	4.0
Meropenem-vaborbactam	Enterobacterales CF, CK, ECC, EC, KP, KV, KA, KO, MM, PM, PV, and SM	841	818	97.3	833	99	41	4.9	1	2.4	1	0.1	6	0.7
Minocycline	*A. baumannii* complex	199	186	93.5	181	91	39	19.6	0	0.0	5	3.3	13	6.5
	Enterobacterales EC, KP, KV, KA, and KO	396	371	93.7	376	94.9	41	10.4	2	4.9	3	0.9	15	3.8
Piperacillin-tazobactam	*A. baumannii* complex	108	102	94.4	103	95.4	56	51.9	1	1.8	0	0.0	4	3.7
	Enterobacterales CF, CK, ECC, EC, KP, KV, KA, KO, MM, PM, PV, and SM	891	830	93.2	855	96	121	13.6	3	2.5	7	0.9	26	2.9
	*P. aeruginosa*	168	155	92.3	158	94	14	8.3	1	7.1	2	1.4	7	4.2
Tobramycin	*P. aeruginosa*	166	155	93.4	162	97.6	14	8.4	0	0.0	1	0.7	3	1.8
Trimethoprim-sulfamethoxazole	Enterobacterales CF, ECC, EC, KP, KV, KA, and KO	522	508	97.3	516	98.9	157	30.1	3	1.9	1	0.3	0	0.0

^*a*^ CA, categorical agreement; R, resistant isolates; mE, minor errors.

^*b*^ Refer to Supplementary table 1 for Selux NGP system FDA-cleared species and antimicrobial agents.

^*c*^ Enterobacterales group includes species: CF, *Citrobacter freundii (complex)*; CK, *Citrobacter koseri*; ECC, *Enterobacter cloacae (complex)*; EC, *Escherichia coli*; KP, *K. pneumoniae*; KV, *K. variicola*; KA, *Klebsiella aerogenes*; KO, *Klebsiella oxytoca*; MM, *Morganella morganii*; PM, *Proteus mirabilis*; PV, *Proteus vulgaris.*

^*d*^ Percentage calculated as follows: %VME = total number VME/total number resistant isolates × 100; %ME = total number ME/total number susceptible isolates × 100; %mE = total number mE/total number isolates × 100.

For both inter- and intra-site reproducibility testing, the best-case calculations were ≥95%, and worst-case calculations were ≥89%, except for penicillin (94.9% inter-site best-case) and erythromycin (93.6% intra-site best-case) as noted [Table S2; reference (17)]. Quantitative cultures from quality control and multicenter study inocula used in the study had mean CFU/mL values of 1.44 × 10^6^ and 9.70 × 10^5^ for GN and GP organisms, respectively. Values varied from 2.10 × 10^5^ to 6.63 × 10^6^ CFU/mL for GN and from 1 × 10^5^ to 4.74 × 10^6^ CFU/mL for GP organisms.

The average TTR for all clinical and challenge isolates is shown in Table 3. The average TTR for all species was 5.65 hours. Results were available within 6.5 hours for 95% of non-*P*. *aeruginosa* organisms.

**TABLE 3:** Average TTR^*a*^

Organism group or species	Total tested	TTR (hour)		
		Mean	Min	Max
*A. baumannii complex*	120	5.66	5.18	7.73
*Citrobacter freundii complex*	64	5.59	5.20	7.27
*Citrobacter koseri*	60	5.66	5.22	7.36
*Enterobacter cloacae complex*	79	5.71	5.19	7.30
*Escherichia coli*	188	5.52	5.17	7.15
*Klebsiella aerogenes*	39	5.59	5.23	6.82
*Klebsiella oxytoca*	31	5.65	5.24	7.18
*Klebsiella pneumoniae, variicola*	141	5.59	5.18	7.15
*Morganella morganii*	63	5.48	5.21	6.74
*Proteus mirabilis*	62	5.53	5.14	6.19
*Proteus vulgaris*	58	5.53	5.20	7.23
*P. aeruginosa*	172	7.04	5.21	10.32
*Serratia marcescens*	63	5.66	5.22	7.30
*Enterococcus faecalis*	110	5.37	5.04	6.24
*Enterococcus faecium*	120	5.47	5.14	8.29
*S. aureus*	156	5.38	5.08	7.09
Coagulase-negative *Staphylococcus* spp.	100	5.63	5.08	8.49

^*a*^ TTR defined as the time panels were placed on the analyzer (Selux NGP assay start) until MIC value was generated.

## DISCUSSION

In this multicenter study, we evaluated the performance of the Selux NGP system for performing AST of non-fastidious GP and GN organisms using FDA breakpoints by comparing it with reference BMD. Except where noted in the Results section, all results met or exceeded acceptable FDA performance metrics, including EA, categorical agreement, and ME and VME rates (17). The Selux NGP system also met ISO 20776–2 performance criteria (22). All species and drug groupings were at or above the 90% EA criteria threshold except for *P. aeruginosa* with ceftazidime-avibactam which was 89.6%. Similarly, bias was below the 30% threshold for all GP, fermentative, and non-fermentative GN species groupings.

The system underwent evaluation using an inoculum range of 0.4–0.6 McFarland units. The reason for choosing this inoculum range was to speed sample setup by easing the burden of precise inoculum preparation for users. This range has now been validated with the results of this study. In addition, CLSI M07 states that a 0.5 McFarland standard results in a range of 1.0–2.0 × 10^8^ CFU/mL of *E*. coli ATCC 25922 (18). The range used in this study, 0.4–0.6 McFarland units corresponding to 1.2–1.8 × 10^8^ CFU/mL of *E*. coli ATCC 25922, is within the range included in the CLSI M07 reference document. This acceptable McFarland range used for colony preparation, as well as for testing organisms of different sizes and shapes, and which have different light scattering properties, likely accounts for the variation in the colony counts seen in this study.

The system underwent evaluation using broad antibiotic dilution ranges for each antibiotic, having at least three concentrations below the current FDA susceptible breakpoint and at least two above the current FDA-resistant breakpoint (Table S1). The availability of these ranges in conjunction with the Breakpoint Change Protocol developed by the manufacturer with FDA during 510(*k*) clearance should facilitate the regulatory review process required for the Selux NGP system to update breakpoints. This is particularly important as the College of American Pathologists (CAP) will require clinical laboratories to demonstrate the use of up-to-date breakpoints (current within 3 years of the most recent update by FDA or other standards development organization used by the laboratory) as part of their laboratory accreditation requirements starting in 2024 (23), though it should be noted that breakpoint updates will have to be validated by each laboratory following CAP guidelines. Each Selux NGP panel also contains a large number of antibiotics, which allows testing of routine, as well as more broad spectrum and newer antimicrobial agents simultaneously, reducing the potential need for reflex testing and delayed results. Finally, the use of 384-well plates allows sufficient area for additional drug inclusion as new agents become available for clinical use.

A limitation of this study is that despite efforts to enrich for non-susceptible isolates, few resistant isolates to ceftaroline, daptomycin, and linezolid were included due to the infrequency of resistance to these agents. The system currently is also only FDA-cleared for non-fastidious organisms.

In conclusion, the Selux NGP system allows for susceptibility testing to begin without a species identification and generates susceptibility results for broad antibiotic menus within 6 hours for most isolates with a high degree of correlation with the BMD standard.

## SUPPLEMENTAL MATERIAL

Supplemental figures and tables jcm.00546-23-s0001.pdf Supplemental figures and tables.

ASM does not own the copyrights to Supplemental Material that may be linked to, or accessed through, an article. The authors have granted ASM a non-exclusive, world-wide license to publish the Supplemental Material files. Please contact the corresponding author directly for reuse.
