# Supplementary material for: Multicenter evaluation of the Selux Next-Generation Phenotyping antimicrobial susceptibility testing system
Source: J Clin Microbiol. 2023 Dec 5;62(1):e00546-23. doi: 10.1128/jcm.00546-23 (PMC10793272; doi:10.1128/jcm.00546-23)
Supplement: Supplemental figures and tables [file jcm.00546-23-s0001.pdf]

Supplementary Information for

Multicenter evaluation of the Selux Next-Generation Phenotyping antimicrobial susceptibility testing system

Supp. Table 1 (pages 2-5). FDA-cleared antimicrobial menus, panel dilution range and breakpoints.

Supp. Table 2 (pages 6-9). Reproducibility study results

Supp. Figure 1 (page 10). Selux NGP Assay workflow

Supplementary Table 1a: FDA-cleared species and antimicrobials for the NGP system gram-positive panel

The Selux gram-positive panel is a quantitative test for the following antimicrobial agents and is a qualitative test for the cefoxitin screen test to predict *mecA*-mediated oxacillin resistance with the specific organisms identified below.

Organism	Ampicillin	Ceftaroline	Clindamycin	Daptomycin	Delafloxacin	Eravacycline	Erythromycin	Linezolid	Levofloxacin	Minocycline	Oxacillin	Penicillin	Trimethoprim	Vancomycin	Cefoxitin Screen
Enterococcus faecalis	x			x	x	x		x	x			x		x	
Enterococcus faecium	x							x	x			x		x	
Staphylococcus aureus		x	x	x	x	x	x	x	x#	x	x	x	x	x	x
**Coagulase-negative *Staphlyococcus* spp.**			x*		x**			x***			x****		x	x	x****

# Methicillin-susceptible *S. aureus*	* *S. epidermidis*<br>** *S. haemolyticus*	*** *S. haemolyticus* and *S. epidermidis*<br>**** *S. lugdunensis*

Supplementary Figure 1b: FDA-cleared antimicrobials for the NGP system gram-positive panel grouped by class

Antimicrobial	Abbreviation	Target Organism(s)	Reporting Range (μ g/mL)	FDA Breakpoints (S/I/R)
**Beta-lactams**				
Ampicillin	AMP	*Enterococcus faecium*<br>*Enterococcus faecalis*	≤0.25 to ≥128	≤8 / ≥16
Oxacillin	OXA	*Staphylococcus aureus*[1]<br>*Staphylococcus lugdunensis*	≤0.03 to ≥32	≤2 / ≥4
Penicillin	PEN	*Enterococcus faecium*<br>*Enterococcus faecalis*<br>*Staphylococcus aureus*[*]	≤0.03 to ≥64	*Enterococcus* spp.: ≤8 / ≥16<br>*S. aureus*: ≤0.12 / ≥0.25
Cefoxitin Screen to predict *mecA*-mediated oxacillin resistance	FOX SCN	*Staphylococcus aureus*<br>*Staphylococcus lugdunensis*	SN or SP[**]	N/A
**Cephalosporins**				
Ceftaroline	CPT	*Staphylococcus aureus*	≤0.06 to ≥32	≤1 / 2 / ≥4
**Lincomycins**				
Clindamycin	CLI	*Staphylococcus aureus*<br>*Staphylococcus epidermidis*	≤0.03 to ≥16	≤0.5 / 1-2 / ≥4
**Macrolides**				
Erythromycin	ERY	*Staphylococcus aureus*	≤0.06 to ≥32	≤0.5 / 1-4 / ≥8
**Cyclic lipopeptides**				
Daptomycin	DAP	*Enterococcus faecalis*<br>*Staphylococcus aureus*	≤0.06 to ≥32	*E. faecalis*: ≤2 / 4 / ≥8<br>*S. aureus*: ≤1 (≥2 NS***)
**Fluoroquinolones**				
Delafloxacin	DFX	*Enterococcus faecalis*<br>*Staphylococcus aureus*<br>*Staphylococcus haemolyticus*	≤0.008 to ≥8	*Enterococcus* spp.:<br>≤0.12 / 0.25 / ≥0.5<br>*Staphylococcus* spp.:<br>≤0.25 / 0.5 / ≥1
Levofloxacin	LVX	*Enterococcus faecium*<br>*Enterococcus faecalis*<br>Methicillin-susceptible *Staphylococcus aureus*	≤0.06 to ≥32	≤2 / 4 / ≥8
**Oxazolidinones**				
Linezolid	LNZ	*Enterococcus faecium*<br>*Enterococcus faecalis*<br>*Staphylococcus aureus*<br>*Staphylococcus epidermidis*<br>*Staphylococcus haemolyticus*	≤0.25 to ≥32	*Enterococcus* spp.:<br>≤2 / 4 / ≥8<br>*Staphylococcus* spp.:<br>≤4 / ≥8
**Tetracyclines**				
Eravacycline	ERV	*Enterococcus faecalis*<br>*Staphylococcus aureus*	≤0.002 to ≥0.5	≤0.06 / ≥0.12
Minocycline	MIN	*Staphylococcus aureus*	≤0.12 to ≥64	≤4 / 8 / ≥16
**Gylcopeptides**				
Vancomycin	VAN	*Enterococcus faecium*<br>*Enterococcus faecalis*<br>*Staphylococcus aureus*<br>*Coagulase-Negative* Staphylococci (CoNS) (including *S. capitis, S. cohnii, S. epidermidis, S. haemolyticus, S. hominis, S. intermedius* group, *S. lugdunensis, S. saprophyticus, S. schleiferi, S. simulans*)	≤0.12 to ≥128	*Enterococcus* spp.:<br>≤4 / 8-16 / ≥32<br>*S. aureus*: ≤2 / 4-8 / ≥16<br>CoNS: ≤4 / 8-16 / ≥32
**Sulfa drugs**				
Trimethoprim	TMP	*Staphylococcus aureus*<br>*Coagulase-Negative* Staphylococci (CoNS) (including *S. capitis, S. haemolyticus, S. saprophyticus, S. simulans*)	≤0.25 to ≥64	≤8 / ≥16

[1]S. aureus reporting range is ≤0.25 to ≥32

*S. aureus reporting range is ≤0.03 to ≥4

**SN, Screen negative; SP, Screen positive

*** NS, nonsusceptible

Supplementary Figure 1c: FDA-cleared species and antimicrobials for the NGP system gram-positive panel

The Selux gram-negative panel is a quantitative test for the following antimicrobial agents with the specific organisms identified below:

Organsim	Amikacin	Amoxicillin-Clavulanate	Ampicillin	Ampicillin-Sulbactam	Aztreonam	Cefazolin	Cefepime	Cefoxitin	Ceftazidime	Ceftazidime-Avibactam	Ceftriaxone	Ciprofloxacin	Eravacycline	Ertapenem	Gentamicin	Imipenem-Relebactam	Levofloxacin	Meropenem	Meropenem-Vaborbactam	Minocycline	Piperacillin-Tazobactam	Tobramycin	Trimethoprim-Sulfamethoxaz
***Acinetobacter baumannii* complex**				x																			
***Citrobacter freundii* complex**							x		x	x	x	x	x	x	x	x	x	x	x				
Citrobacter koseri							x		x	x	x	x		x	x	x	x	x	x		x		
***Enterobacter cloacae* complex**							x		x	x		x	x		x	x	x	x	x				x
Escherichia coli		x	x	x	x	x	x	x	x	x	x	x	x	x	x	x	x	x	x		x		
Klebsiella aerogenes							x		x	x	x	x			x		x		x	x			x
Klebsiella oxytoca		x		x			x	x	x	x	x	x	x	x	x	x	x	x	x	x			x
Klebsiella pneumoniae		x		x		x	x	x	x	x	x	x		x	x		x	x	x	x	x		x
Morganella morganii							x	x		x		x		x			x	x	x				
Proteus mirabilis		x	x	x			x		x	x	x	x		x	x		x	x			x		
Proteus vulgaris				x			x		x	x		x		x	x		x	x			x		
Serratia marcescens							x		x	x				x	x		x	x	x				
Psuedomonas aeruginosa	x									x		x			x	x		x				x	

Supplementary Figure 1d: FDA-cleared antimicrobials for the NGP System gram-negative panel grouped by class

Antimicrobial	Abbreviation	Targeted Organism	Reporting Range (μ g/mL)	FDA Breakpoints (S/I/R)
**Monobactams**				
Aztreonam	ATM	Enterobacterales	≤0.03 to ≥128	≤4 / 8 / ≥16
**Beta-lactams**				
Ampicillin	AMP	Enterobacterales	≤0.25 to ≥128	≤8 / 16 / ≥32
**Cephalosporins**				
Cefazolin (1st gen)	CFZ	Enterobacterales	≤0.12 to ≥128	≤1 / 2 / ≥4
Cefoxitin (2nd gen)	FOX	Enterobacterales	≤1 to ≥128	≤4 / 8 / ≥16
Ceftazidime (3rd gen)	CAZ	Enterobacterales	≤0.25 to ≥64	≤4 / 8 / ≥16
Ceftriaxone (3rd gen)	CRO	Enterobacterales	≤0.25 to ≥32	≤1 / 2 / ≥4
Cefepime (4th gen)	FEP	Enterobacterales	≤0.25 to ≥128	≤2 / 4-8 / ≥16
**Carbapenems**				
Ertapenem	ETP	Enterobacterales	≤0.03 to ≥16	≤0.5 / 1 / ≥2
Imipenem	IMI	*Acinetobacter baumannii* complex	≤0.016 to ≥32	≤2/ 4 / ≥8
Meropenem	MEM	Enterobacterales<br>*Pseudomonas aeruginosa*	≤0.12 to ≥64	Enterobacterales:<br>≤1 / 2 / ≥4<br>*Pseudomonas aeruginosa:*<br>≤2 / 4 / ≥8
**Beta-lactam/Inhibitors**				
Ampicillin-Sulbactam	SAM	*Acinetobacter baumannii* complex<br>Enterobacterales	≤0.5 to ≥128	≤8 / 16 / ≥32
Amoxicillin-Clavulanate	AMC	Enterobacterales	≤0.5 to ≥128	≤8 / 16 / ≥32
Piperacillin-Tazobactam	TZP	Enterobacterales	≤0.25 to ≥512	≤16 / 32-64 / ≥128
**Beta-lactam/Inhibitor next-generation**				
Ceftazidime-Avibactam	CZA	Enterobacterales<br>*Pseudomonas aeruginosa*	≤0.12 to ≥64	≤8 / ≥16
Imipenem-Relebactam	IMR	Enterobacterales<br>*Pseudomonas aeruginosa*	≤0.03 to ≥128	Enterobacterales:<br>≤1 / 2 / ≥4<br>*Pseudomonas aeruginosa:*<br>≤2 / 4 / ≥8
Meropenem-Vaborbactam	MEV	Enterobacterales	≤0.06 to ≥64	≤4 / 8 / ≥16
**Fluoroquinolones**				
Levofloxacin	LVX	Enterobacterales	≤0.06 to ≥32	≤0.5 / 1 / ≥2
Ciprofloxacin	CIP	Enterobacterales<br>*Pseudomonas aeruginosa*	≤0.03 to ≥16	Enterobacterales:<br>≤0.25 / 0.5 / ≥1<br>*Pseudomonas aeruginosa:*<br>≤0.5 / 1 / ≥2
**Tetracyclines**				
Eravacycline	ERV	Enterobacterales	≤0.016 to ≥4	≤0.5 / -
Minocycline	MIN	Enterobacterales	≤0.25 to ≥64	≤4 / 8 / ≥16
**Aminoglycosides**				
Amikacin	AMK	*Pseudomonas aeruginosa*	≤0.12 to ≥256	≤16 / 32 / ≥64
Gentamicin	GEN	Enterobacterales<br>*Pseudomonas aeruginosa*	≤0.06 to ≥64	≤4 / 8 / ≥16
Tobramycin	TOB	*Pseudomonas aeruginosa*	≤0.12 to ≥128	≤4 / 8 / ≥16
**Sulfa drugs**				
Trimethoprim-Sulfamethoxazole	SXT	Enterobacterales	≤0.12 to ≥32	≤2 / ≥4

Supplementary Table 2a Gram-positive Inter-site reproducibility*[a]

Selux AST System Inter-site Reproducibility				
	All organisms (combined)		Indicated organisms only^	
**Antimicrobial**	**Best-case (%)**	**Worst case (%)**	**Best-case (%)**	**Worst case (%)**
Ampicillin	72/75 (96%)	72/75 (96%)	68/69 (98.6%)	68/69 (98.6%)
Cefoxitin screen	74/75 (98.7%)	74/75 (98.7%)	74/75 (98.7%)	74/75 (98.7%)
Ceftaroline	74/75 (98.7%)	74/75 (98.7%)	74/75 (98.7%)	74/75 (98.7%)
Clindamycin	78/81 (96.3%)	78/81 (96.3%)	63/66 (95.5%)	63/66 (95.5%)
Daptomycin	77/78 (98.7%)	77/78 (98.7%)	77/78 (98.6%)	77/78 (98.6%)
Delafloxacin	144/144 (100%)	144/144 (100%)	144/144 (100%)	144/144 (100%)
Eravacycline	78/78 (100%)	78/78 (100%)	78/78 (100%)	78/78 (100%)
Erythromycin	141/144 (97.9%)	136/144 (94.4%)	141/144 (97.9%)	136/144 (94.4%)
Levofloxacin	76/78 (97.4%)	76/78 (97.4%)	76/78 (97.4%)	76/78 (97.4%)
Linezolid	77/78 (98.7%)	77/78 (98.7%)	56/57 (98.2%)	56/57 (98.2%)
Minocycline	73/75 (97.3%)	73/75 (97.3%)	64/66 (97.0%)	64/66 (97.0%)
Oxacillin	76/78 (97.4%)	75/78 (96.2%)	76/78 (97.4%)	75/78 (96.2%)
Penicillin	74/78 (94.9%)	70/78 (89.7%)	74/78 (94.9%)	70/78 (89.7%)
Trimethoprim	77/81 (95.1%)	74/81 (91.4%)	77/81 (95.1%)	74/81 (91.4%)
Vancomycin	79/80 (98.8%)	75/80 (93.8%)	79/80 (98.8%)	75/80 (93.8%)

*Best-case performance assumes off-scale results were within essential agreement and worst-case performance assumes off-scale results were not within essential agreement.

[a]Refer to Supp. Table 1 for NGP System FDA-cleared species and antimicrobial agents

^Indicated organisms are those listed in the reference listed drug (RLD) label

Supplementary Table 2b Gram-positive Intra-site reproducibility*[a]

Selux AST System Intra-site Reproducibility				
	All organisms (combined)		Indicated organisms only^	
**Antimicrobial**	**Best-case (%)**	**Worst case (%)**	**Best-case (%)**	**Worst case (%)**
Ampicillin	45/45 (100%)	45/45 (100%)	45/45 (100%)	45/45 (100%)
Cefoxitin screen	47/47 (100%)	47/47 (100%)	47/47 (100%)	47/47 (100%)
Ceftaroline	48/49 (98.0%)	48/49 (98.0%)	48/49 (98.0%)	48/49 (98.0%)
Clindamycin	54/54 (100%)	54/54 (100%)	36/36 (100%)	36/36 (100%)
Daptomycin	74/74 (100%)	74/74 (100%)	65/65 (100%)	65/65 (100%)
Eravacycline	103/103 (100%)	99/103 (96.1%)	103/103 (100%)	99/103 (96.1%)
Erythromycin[1]	44/47 (93.6%)	42/47 (89.4%)	44/47 (93.6%)	42/47 (89.4%)
Linezolid	62/63 (98.4%)	62/63 (98.4%)	53/54 (98.1%)	53/54 (98.1%)
Oxacillin	62/65 (95.4%)	62/65 (95.4%)	62/65 (95.4%)	62/65 (95.4%)
Vancomycin	83/83 (100%)	79/83 (95.2%)	83/83 (100%)	79/83 (95.2%)

*Best-case performance assumes off-scale results were within essential agreement and worst-case performance assumes off-scale results were not within essential agreement.

[a]Refer to Supp. Table 1 for NGP System FDA-cleared species and antimicrobial agents

^Indicated organisms are those listed in the reference listed drug (RLD) label

[1]The best-case intra-site reproducibility for erythromycin was 93.6%, which is <95%; however, reproducibility testing at two other sites were 100%

Supplementary Table 2c Gram-negative Inter-site reproducibility*[a]

Selux AST System Inter-site Reproducibility				
	All organisms (combined)		Indicated organisms only^	
Antimicrobial	Best-case (%)	Worst case (%)	Best-case (%)	Worst case (%)
Amikacin	98/104 (94.2%)	98/104 (94.2%)	92/98 (93.9%)	92/98 (93.9%)
Amoxicillin-Clavulanate	71/72 (98.6%)	70/72 (97.2%)	71/72 (98.6%)	70/72 (97.2%)
Ampicillin	72/75 (96.0%)	72/75 (96.0%)	68/69 (98.6%)	68/69 (98.6%)
Ampicillin-Sulbactam	74/75 (98.7%)	74/75 (98.7%)	66/66 (100%)	66/66 (100%)
Aztreonam	77/78 (98.7%)	75/78 (96.2%)	77/78 (98.7%)	75/78 (96.2%)
Cefazolin	77/81 (95.1%)	76/81 (93.8%)	68/72 (94.4%)	67/72 (93.1%)
Cefepime	77/78 (98.7%)	76/78 (97.4%)	77/78 (98.7%)	76/78 (97.4%)
Cefoxitin	70/72 (97.2%)	69/72 (95.8%)	70/72 (97.2%)	69/72 (95.8%)
Ceftazidime	77/81 (95.1%)	71/81 (87.7%)	77/81 (95.1%)	71/81 (87.7%)
Ceftazidime-Avibactam	140/144 (97.2%)	139/144 (96.5%)	140/144 (97.2%)	139/144 (96.5%)
Ceftriaxone	140/141 (99.3%)	140/141 (99.3%)	140/141 (99.3%)	140/141 (99.3%)
Ciprofloxacin	74/75 (98.7%)	71/75 (94.7%)	74/75 (98.7%)	71/75 (94.7%)
Eravacycline	78/78 (100%)	78/78 (100%)	78/78 (100%)	78/78 (100%)
Ertapenem	143/145 (98.6%)	143/145 (98.6%)	143/145 (98.6%)	143/145 (98.6%)
Gentamicin	77/80 (96.3%)	77/80 (96.3%)	77/80 (96.3%)	77/80 (96.3%)
Imipenem	145/149 (97.3%)	140/149 (94.0%)	145/149 (97.3%)	140/149 (94.0%)
Imipenem-Relebactam	72/75 (96.0%)	72/75 (96.0%)	72/75 (96.0%)	72/75 (96.0%)
Levofloxacin	76/78 (97.4%)	76/78 (97.4%)	76/78 (97.4%)	76/78 (97.4%)
Meropenem	75/78 (96.2%)	73/78 (93.6%)	70/72 (97.2%)	68/72 (94.4%)
Meropenem-Vaborbactam	142/145 (97.9%)	132/145 (91.0%)	133/143 (93.0%)	133/143 (93.0%)
Minocycline	73/75 (97.3%)	73/75 (97.3%)	64/66 (97.3%)	64/66 (97.3%)
Piperacillin-Tazobactam	72/75 (96.0%)	69/75 (92.0%)	63/66 (95.5%)	60/66 (90.9%)
Tobramycin	74/78 (94.9%)	73/78 (93.6%)	74/78 (94.9%)	73/78 (93.6%)
Trimethoprim-Sulfamethoxazole	143/148 (96.6%)	142/148 (95.9%)	143/148 (96.6%)	142/148 (95.9%)

*Best-case performance assumes off-scale results were within essential agreement and worst-case performance assumes off-scale results were not within essential agreement.

[a]Refer to Supp. Table 1 for NGP System FDA-cleared species and antimicrobial agents

^Indicated organisms are those listed in the reference listed drug (RLD) label

Supplementary Table 2d Gram-negative Intra-site reproducibility*[a]

Selux AST System Intra-site Reproducibility				
	All organisms (combined)		Indicated organisms only^	
**Antimicrobial**	**Best-case (%)**	**Worst case (%)**	**Best-case (%)**	**Worst case (%)**
Amoxicillin-Clavulanate	63/63 (100%)	63/63 (100%)	63/63 (100%)	63/63 (100%)
Ampicillin	45/45 (100%)	45/45 (100%)	45/45 (100%)	45/45 (100%)
Ampicillin-Sulbactam	72/72 (100%)	72/72 (100%)	72/72 (100%)	72/72 (100%)
Aztreonam	71/74 (95.9%)	68/74 (91.9%)	71/74 (95.9%)	68/74 (91.9%)
Cefazolin	61/63 (96.8%)	61/63 (96.8%)	61/63 (96.8%)	61/63 (96.8%)
Cefepime	45/47 (95.7%)	45/47 (95.7%)	45/47 (95.7%)	45/47 (95.7%)
Cefoxitin	54/54 (100%)	54/54 (100%)	54/54 (100%)	54/54 (100%)
Ceftazidime	92/93 (98.9%)	75/93 (80.6%)	92/93 (98.9%)	75/93 (80.6%)
Ceftazidime-Avibactam	44/47 (93.6%)	44/47 (93.6%)	44/47 (93.6%)	44/47 (93.6%)
Eravacycline	103/103 (100%)	99/103 (96.1%)	103/103 (100%)	99/103 (96.1%)
Gentamicin	116/121 (95.9%)	116/121 (95.9%)	116/121 (95.9%)	116/121 (95.9%)
Imipenem-Relebactam	72/76 (94.7%)	72/76 (94.7%)	72/76 (94.7%)	72/76 (94.7%)
Levofloxacin	179/181 (98.9%)	169/181 (93.4%)	179/181 (98.9%)	169/181 (93.4%)
Meropenem	57/58 (98.3%)	56/58 (96.6%)	57/58 (98.3%)	56/58 (96.6%)
Meropenem-Vaborbactam	45/47 (95.7%)	43/47 (91.5%)	45/47 (95.7%)	43/47 (91.5%)
Piperacillin-Tazobactam	54/56 (96.4%)	54/56 (96.4%)	54/56 (96.4%)	54/56 (96.4%)
Trimethoprim-Sulfamethoxazole	62/65 (95.4%)	61/65 (93.8%)	62/65 (95.4%)	61/65 (93.8%)

*Best-case performance assumes off-scale results were within essential agreement and worst-case performance assumes off-scale results were not within essential agreement.

[a]Refer to Supp. Table 1 for NGP System FDA-cleared species and antimicrobial agents

^Indicated organisms are those listed in the reference listed drug (RLD) label

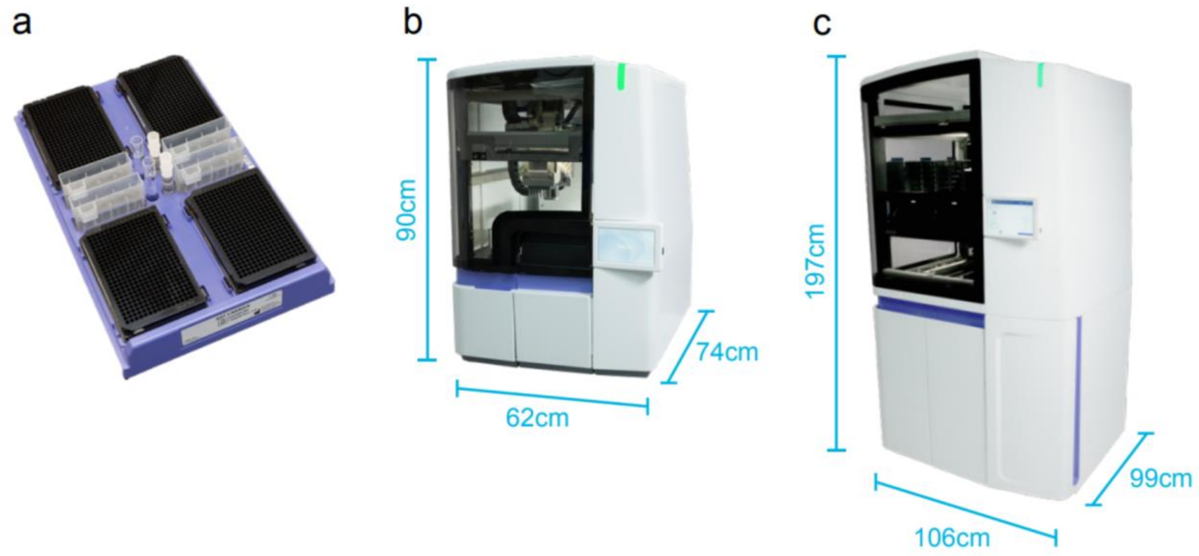

Supplementary Figure 1. *Selux NGP Assay workflow*.

(a) Sample prep Carrier. Users begin by selecting the organism's gram type and appropriate Selux AST panel. Users then prepare a 0.4-0.6 McFarland inoculum and load it with the appropriate panel onto the carrier. Up to 4 samples can be loaded onto each carrier.

(b) Users next move carriers to the inoculator, which dilutes the inoculum into growth media (1:200 dilution into cation-adjusted Mueller-Hinton broth) and inoculates each of the 1-4 panels on the carrier at a rate of approximately 3.5 minutes per panel (multiple inoculators may be used per analyzer). The inoculator aliquots 50 µL into panel wells. Precise control over the number of bacteria aliquoted into each well is essential for rapid AST to be accurate. As the growth in each dilution is determined relative to other dilutions, and since there are few doubling cycles possible with rapid AST, ensuring starting concentrations are equivalent is paramount.

(c) When inoculation is complete the carrier is returned to the user for loading into the analyzer, a random-access system that fully automates AST processing. After AST processing is complete and the sample ID is available, the panel data are processed by the algorithm. The system can be configured to automatically release AST results to the laboratory information system (LIS). The system can interface with ASTM or HL7 formatted LIS interfaced systems through middleware. The NGP system can also interface with MALDI-TOF systems via the laboratory's local network, and any future changes in species nomenclature can be accommodated by NGP system software updates.
